# Strategic Processing of Chinese Young English Language Learners in an International Standardized English Language Test

**DOI:** 10.3389/fpsyg.2018.01020

**Published:** 2018-06-27

**Authors:** Feifei Han

**Affiliations:** Centre for Research on Learning and Innovation, Sydney School of Education and Social Work, Faculty of Arts and Social Sciences, The University of Sydney, Sydney, NSW, Australia

**Keywords:** strategic processing, Chinese young English language learners, international standardized test, Flyers test, cognitive and metacognitive strategies

## Abstract

Strategic competence is acknowledged to be able to explain variations in language test performance. Research with adult language test-takers has shown that strategic competence has dual components: strategic knowledge and strategic processing. Of the two components, strategic processing, which is state-like, unstable, and tends to fluctuate from contexts to contexts, is more closely related to language test performance. To date, none of the existing studies investigates strategic processing with children English language learners (ELLs) and explores the relationship between strategic processing in all the four skills of language learning and the test performance. Addressing these gaps, the current study examined the nature of strategic processing in listening, reading and writing, and speaking of 138 Chinese young ELLs in an international standardized English language test – Cambridge Young Learners English Tests – Flyers test. The three questionnaires regarding strategic processing were administered to the participants immediately following the completion of the test. The confirmatory factor analyses verified that the strategic processing construct in the four skills comprised of a cognitive and a metacognitive dimensions, which resembles the strategic processing of the adult language test-takers. The participants adopted significantly more metacognitive than cognitive strategies consistently in the three sections of the test, possibly due to the status of the test. Both cognitive and metacognitive strategic processing were moderately related to the test performance, explaining from 7 to 31% of the variance in the total shields of the test. Across the four skills, high-performing test-takers used both cognitive and metacognitive strategies more frequently than the moderate- and low-performing test-takers, even though whether such differences were due to their richer strategic knowledge or processing skills was unknown. The study contributes to strategic processing in language testing literature and also provides practical implications for English trainers of the young ELLs in China.

## Introduction

With an increasingly wide-spread of English language in every domain worldwide, English proficiency tests play a powerful role not only in critical decision making processes, such as in deciding whether a student can enter a university, whether an individual can be successful in job hunting, or whether an applicant can obtain a permanent residency in an English speaking country; but also serves to motivate/demotivate learners to sustain their efforts in learning English (Zhang et al., 2010). Consequently, factors which impact on English language test performance have become a heated issue for discussion among language testing researchers, and English instructors.

For a long time, language testing/assessment specialists have been making great endeavor into assessing foreign language (FL) performance validly and have proposed different theoretical models in an attempt to explain observed language test performance/scores (e.g., Hymes, 1972; Canale and Swain, 1980; Bachman, 1990; Bachman and Palmer, 1996, 2010). The researchers concur that even though language ability is the major factor explaining success in language test performance/scores, a number of non-linguistic factors may also contribute to such performance (Bachman, 2000; Bachman and Palmer, 2010; Purpura, 2014; Phakiti, 2016). Strategic competence is one of the non-linguistic factors, which have been researched among adult (e.g., Purpura, 1997, 1998, 1999; Phakiti, 2003a,b, 2006, 2008a,b) and adolescent (e.g., Nikolov, 2006) test-takers. With an increasing trend that English learning commences at an earlier age and more and more English language tests are designed for child English language learners (ELLs), research is needed to investigate strategic competence among the vast number of young children population. To fill the gap in the literature, the current study will make the first attempt to investigate strategic processing (i.e., one aspect of strategic competence) in Cambridge English: Flyers test – an international standardized English language test among Chinese child ELLs. The following part reviews the relevant literature.

### Literature Review and Research Questions

#### Strategic Competence in Language Testing

Bachman and Palmer (1996, 2010) list four major factors which may impact on language test performance: (1) communicative language ability (CLA); (2) test method facets, including testing environments, test rubrics, test delivery modes, and the nature of the responses; (3) test-takers’ individual characteristics, such as background, personal attributes, social psychological factors, and cognitive abilities; and (4) random measurement error. Within the CLA, Bachman and Palmer further attempt to separate linguistic and non-linguistic elements in their well-known hypothesized model – CLA model. The two major aspects included in the CLA model are the linguistic aspect (e.g., language competence) and the non-linguistic aspect, of which strategic competence is deemed to be important (McNamara, 1996; Phakiti, 2016). Bachman and Palmer define strategic competence as “the mental capacity for implementing the components of language competence in contextualized communicative language use” (p. 106). It is a higher-order cognitive mechanism which orchestrates a set of strategies to regulate an individual’s online cognitive processing (e.g., coordinating strategies), linguistic processing (e.g., mental searching of linguistic elements), and psychological processing (e.g., regulating one’s affect and anxiety) through one’s metacognition by in order to accomplish a communicative goal (Phakiti, 2008a,b, 2016).

McNamara (1996) and Phakiti (2008a) postulate that strategic competence encompasses two components: strategic knowledge and strategic processing. While strategic knowledge is considered being relatively stable and stored in the long-term memory (Han and Stevenson, 2008; Phakiti, 2008a,b; Han, 2012, 2013; Wang and Han, 2017); strategic processing is related to the online information processing and heavily hinges upon the contexts, hence relatively unstable (Schmidt, 2001; Cohen, 2007). The postulation of the construction of strategic competence has also been validated and the stability of the construct has also been established in Phakiti’s, (2008a,b) longitudinal studies, which showed a clear distinction between strategic knowledge and strategic processing in a customarily designed FL reading testing context. For strategic processing to be executed successfully, merely having strategic knowledge is only a necessary but not a sufficient condition (Han and Stevenson, 2008; Han, 2012, 2013, 2017b; Wang and Han, 2017), as the online processing is likely to be contingent upon a number of factors, such as task difficulty level at hand, a person’s working memory capacity, and an individual’s motivation to the tasks and contexts (Schraw, 1998, 2001; Robinson, 2001; Phakiti, 2008a). In the language tests, for instance, the same test-taker may adjust how they orchestrate test-taking strategies depending on the important of the test to him/her (e.g., when he/she attends an international standard language test vs. a local language test organized by the school).

#### Operationalization of Test-Taking Strategies

Test-taking strategies are a common set of “learner strategies applied to the area of assessment” when solving language test tasks (Cohen, 1994, p. 119). Essentially, test-taking strategies belong to language learner strategies (Bachman and Cohen, 1998; Cohen, 1998b; Phakiti, 2003a, 2016; Nikolov, 2006; Radwan, 2011); which have long been difficult to define and to operationalize (Dörnyei and Skehan, 2002; Nikolov, 2006). In addition, a wide range of taxonomies have been proposed (e.g., Bialystok, 1990; O’Malley and Chamot, 1990; Oxford, 1990; Dörnyei and Scott, 1997; Cohen, 1998a; McDonough, 1999; Macaro, 2001a,b, 2006; Cohen and Macaro, 2007). However, researchers have reached some degree of consensus that consciousness is critical to define strategies (Cohen, 1998a, 2007). For “strategies” to be meaningful, the strategic behaviors and processes have to be taken place within at least the peripheral attention if not within the focal attention of one’s working memory (Ellis, 1994; Schmidt, 2001; Cohen, 2007; Phakiti, 2008b). This means that unconscious processing and behaviors cannot be qualified as strategies, and intentionality and consciousness are essential characteristics of strategies (Cohen, 1998a, 2007). Concurring with Cohen, Oxford (2011) states that “when strategy use is developed into an automatic operation (proceduralized) through repeated practice, it is no longer a strategy but an unconscious habit” (p. 51). Similarly, Ellis (1994) agrees that if strategies have developed to the state of automaticity that learners are unaware of them or unable to describe them, they may not be called as strategies any more. These definitions have in common that they all highlight *awareness and deliberation*, which are the essence to distinguish strategies from other cognitive processing, such as skills – automatic and unconscious cognitive operations (Petric and Czarl, 2003). In this study *awareness and deliberation* were taken into consideration when operationalizing test-taking strategies, which allowed strategies to be measured through learners’ self-report, such as responding to close-ended questionnaires.

#### Strategic Competence and Language Test Performance

Research looks into the relationship between strategic competence and language test performance falls in two categories. While one category is concerned with the relationship between strategic knowledge and test performance, the other deals with strategic processing and performance. Most of the earlier studies examined strategic knowledge because these studies used questionnaires with items written in simple present tense, which measures knowledge *per se*; and the distribution of the questionnaires proceeded the tests, this did not allow learners to reflect upon their strategy use in the tests.

For instance, in three related studies, Purpura (1997, 1998, 1999) administered an 80-item questionnaire, which evaluated knowledge of language use strategy and a University of Cambridge Local Examinations Syndicate’s First Certificate in English Anchor Test to 1,382 test-takers. Using structural equation model (SEM), Purpura (1997) identified a multidimensional structure for the cognitive strategies, which consisted of comprehending, memorizing, and retrieving subscales, but a unidimensionality for the metacognitive strategies. He also found that while knowledge of cognitive strategies had direct and positive impacts on the test performance, metacognitive strategies were only indirectly related to the test performance through direct and positive effects on all the three subscales of cognitive strategies. Still within this project, Purpura (1998) also reported that the effects of metacognitive strategies on cognitive strategies were variant across the high- and low-ability groups: metacognitive strategies had a stronger total effect on cognitive strategies for the low-ability test-takers than their high-ability counterparts. Concerning the effects of metacognitive strategies on the test performance, the results showed that while metacognitive strategies had no direct effect on the test performance among the high-ability test-takers, it had significant, though small, effects on the lexico-grammar and reading sections of the test. Regarding the effects of cognitive strategies on the test performance, Purpura observed that retrieving strategies positively affected all the sections of the test among the low-ability group, whereas they only affected word formation section among the high-ability group. Although standing as pioneering research on strategic knowledge in language testing, a closer examination of the items in the questionnaire shows inappropriateness of some items, which are language learning strategies (e.g., I try to connect what I am learning with what I already know) rather than language use or language test-taking strategies. Therefore, the effects of the strategies on the test performance may not be reliable. A further limitation is that having the knowledge about strategy use does not mean that these participants truly used them in the test.

Refining Purpura’s (1997, 1998, 1999) questionnaires, Song (2005) used 27 items to measure 159 adult ELLs’ knowledge of cognitive strategies and 16 items of metacognitive strategies and examined the relationship between strategic knowledge and test performance of the Michigan English Language Assessment Battery (MELAB). Song identified six dimensions of knowledge of cognitive strategies, namely repeating/conforming information, writing strategies, practice strategies, generating, applying rules, and linking with prior knowledge, and three dimensions of knowledge of metacognitive strategies, namely evaluating, monitoring, and assessing. Using regression analysis, she found that the different subscales made different contributions to different parts of the test, explaining 21.40% for writing, 17.20% for listening, and 12.50% for grammar, cloze, vocabulary and reading. Similar to Purpura’s studies, the limitation of Song’s study is that the participants’ knowledge of strategies do not equate to their use of these strategies in the test, which tends to be influenced by a number of factors, such as test difficulty, learners’ linguistic competence and linguistic processing efficiency, and their perceptions of status of the test (Schraw, 1998, 2001; Robinson, 2001; Phakiti, 2008a).

Phakiti (2003a,b, 2006) started to examine strategic processing in language testing in a series of studies. He wrote questionnaires in simple past tense and administered them immediately after completion of the tests and asked the learners to report the frequency of using cognitive and metacognitive strategies in the test they had just took part in. In his studies, Phakiti conceptualized strategic processing as two-dimensional construct, encompassing a cognitive and a metacognitive strategic processing component. Defining the two in the language testing context, He defined cognitive strategic processing as test-takers’ conscious behaviors to understand and complete a test, which is mainly used to comprehend, memorize, and retrieve information during the test; whereas metacognitive strategic processing is test-takers’ conscious behaviors that regulate cognitive strategic processing and is operated to plan, monitor, and evaluate past, current, and future actions in the processes of completing a test. In these studies, Phakiti examined the relationship between strategic processing and language test performance in both cross-sectional and longitudinal studies.

In a cross-sectional study, Phakiti (2003b) found that both cognitive (*r* = 0.39) and metacognitive strategic processing (*r* = 0.47) were positively and moderately associated with the test performance on a final-term English reading examination among 384 Thai university ELLs. He also found that high-achieving test-takers used significantly more metacognitive strategies than moderate-achieving test-takers, who in turn, adopted more metacognitive strategies than low-achieving learners; whereas there was no significant difference between high- and moderate-achieving learners in terms of using cognitive strategies.

In the longitudinal studies, in addition to the relationship between test performance and strategic processing, Phakiti (2007, 2008a,b) also investigated (1) the construct of strategic competence and (2) the interrelatedness between knowledge, processing, and test scores. Among 561 Thai university ELLs, the studies measured strategic knowledge using a trait strategy questionnaire, whose items were written in simple present tense without specific contexts; and the information of strategic processing was gathered using a state strategy questionnaire, in which all the items were constructed in simple past tense and were restricted in either a mid-term or a final reading test. The SEM measurement model demonstrated that strategic knowledge and processing were clearly distinguished even though the two are highly related. Consistently across the two occasions of the tests, strategic processing was found to be composed of two factors: cognitive and metacognitive processing. The studies further showed that while cognitive strategic processing measured in the two tests directly affected performance on the two tests respectively, albeit with varying effect size; metacognitive strategic processing, cognitive strategic knowledge, and metacognitive strategic knowledge only indirectly contributed to the test scores. The results also demonstrated that strategic processing was more strongly related to the test scores than strategic knowledge did. This also empirically substantiated that the examination of the relationship between strategic processing and test performance is more meaningful than that between strategic knowledge and performance, because the former is what test-takers have done in a test rather than what they know about these strategies, which may or may not be used in a particular test.

However, these studies also reveal some research gaps which need to be addressed. The construct of strategic processing in these studies were restricted in only one skill of a language test, and the tests were also non-standardized and locally constructed in a Thai university. Further research should extend into language tests of international standards and include all the four skills to show if the construct of strategic processing is consistent across the four skills. In addition, research should also be conducted with different populations, especially with children test-takers to see whether the strategic processing components and their relationship with test scores obtained among adult population is applicable to young ELLs. All these limitations will be addressed in the current study.

#### The Current Study and Research Questions

The current study will fill the above research gaps in the literature of strategic processing in language testing. First, it will examine child ELLs’ strategic processing in language testing, which has both theoretical and practical significance. Theoretically, the young ELLs are experiencing cognitive development, hence their strategic repertoire are presumably not as mature as those of the adult ELLs. This may not only affect how they utilize strategic knowledge to complete language tests as reflected by the nature of strategic processing, but may also influence the relationship between strategic processing and language test performance. Practical speaking, investigation of strategic processing among young ELLs may help instructors identify young ELLs’ problems in strategic processing, and design relevant training programs to address these problems.

Secondly, the current study will examine strategic processing in the four skills of language use rather than merely one skill as in the existing studies (e.g., Phakiti, 2003a,b, 2006, 2008a,b). This may reveal whether the nature of strategic processing, and its relation to performance differ in different skills. In addition, the study will examine strategic processing in an international standardized English language test rather than locally constructed tests.

To be specific, the current study will investigate Chinese child ELLs’ strategic processing when they undertake listening, reading and writing, and speaking sections of Cambridge English: Flyers test. The study addresses the three research questions:

(1) What is the nature of strategic processing in listening, reading and writing, and speaking of the Cambridge English: Flyers test among Chinese young ELLs?
(2) To what extent do Chinese young ELLs’ strategic processing in the listening, reading and writing, and speaking relate to the test performance in each section of and the total score of the Cambridge English: Flyers test?
(3) To what extent do high- and low-performing test-takers operate cognitive and metacognitive strategic processing differently in the listening, reading and writing, and speaking of the Cambridge English: Flyers test?

## Materials and Methods

### The Research Design

The current research adopted a quantitative method using questionnaires, which was decided based on the research setting. As the study was conducted in an international standardized English language test, using qualitative methods, such as think-aloud during the test was not possible. Using questionnaires has a number of advantages, such as being less intrusive to the participants than using the think-aloud method; and easiness of administering to a large population, which may make the results more generalizable than qualitative studies. Furthermore, answering questionnaires does not require much time and cognitive burden of the participants so that they would not feel tired after long time concentration on completing an important test. However, using questionnaires to elicit strategic processing has also received critiques that the responses to the questionnaires may not necessarily reflect the online processing (Han, 2017a). To minimize this drawback, the shorter the time interval between answering questionnaires and completion of the test, the data are more reliable. Therefore, participants were asked to respond to the questionnaires immediately after the test by reflecting upon what strategies they had just adopted when completing each section of the Flyers test (The details of questionnaire construction and the analyses of the questionnaires to answer research questions are described below in Section “Materials and Materials” and Section “Data Analysis” respectively).

### Research Setting and Participants

The study was carried out in an international standardized English test for young ELLs, known as Cambridge English: Flyers test (Flyers test hereafter). The Flyers test is the third of a suite of three Cambridge Young Learners English Tests, which are specially designed for children in primary and lower-secondary school. The Flyers test is designed to examine children’s English proficiency of everyday written and spoken English. According to Cambridge English^1^, the difficulty level of the test is level 2 in the Common European Framework of Reference for Languages. Consisted of three sections, namely listening, reading and writing, and speaking, the test is paper-based and lasts for approximately 1 h and 15 min. The test aims to examine young ELLs’ ability to (1) understand simple written English, (2) to communicate with English speakers who speak slowly and clearly in realistic everyday situations, and (3) to understand and use basic English phrases and expressions. One of the main aims for preparing and taking the Flyers test is to motivate learners, therefore, all the test-takers will receive a Cambridge English certificate to recognize their English learning achievements, hence, the test does not set a cut-off score for pass or fail. All the test-takers’ performance in each section receives a raw score, which is then translated into a shield (from 1 to 5). The purpose of using shields is “to equate different test versions” and “the shield score boundaries are set so that all candidates’ results relate to the same scale of achievement.” “This means, for example, a shield boundary may be set at a slightly different raw score across versions” (Cambridge English Language Assessment, 2018, p. 3). Adding the shields of each section in the test gives a total shields for test-takers. **Table 1** summarizes the test format of each section in the Flyers test, including the number of parts and questions, time allowance, and the maximum achievable marks (see the sample test and detailed descriptions of the test format of the Flyers test at http://www.cambridgeenglish.org/exams-and-tests/flyers/test-format/. The participants were 138 Chinese young ELLs who sat the Flyers test in a test center in China to voluntarily participate the study. The descriptive statistics of the participants’ shields, including minimum (min.), maximum (max.), Means (Ms), and Standard Deviations (SDs) of the shields for each section and the total shields are displayed in **Table 2**.

**Table 1:** Test format of the Flyers test.

Section	Contents	Time allowed	Maximum marks
Listening	5 parts/25 questions	About 25 min	5 shields
Reading and writing	7 parts/50 questions	40 min	5 shields
Speaking	4 parts	About 7–9 min	5 shields


**Table 2:** Descriptive statistics of the shields of the Flyers test.

Section	Minimum	Maximum	*M*	*SD*
Listening	1.00	5.00	3.07	0.96
Reading and writing	1.00	5.00	3.36	0.92
Speaking	4.00	5.00	4.96	0.21
Total	5.00	15.00	10.46	2.12


### Materials

#### The Strategic Processing Questionnaires

To elicit the participants’ strategic processing in completing the Flyers test, three questionnaires were used, and each of them corresponded to each part of the Flyers test. The three questionnaires were the Strategic Processing in Listening Questionnaire, Strategic Processing in Reading and Writing Questionnaire and Strategic Processing in Speaking Questionnaire. As no research has been conducted on strategic processing in language testing with young ELLs, the questionnaires were customarily constructed by drawing on the literature and questionnaires of language learning and use strategies in the four skills (e.g., O’Malley and Chamot, 1990; Oxford, 1990; Purpura, 1997, 1998, 1999; Phakiti, 2003a,b, 2006, 2008a,b; Song, 2005; Vandergrift, 2005; Radwan, 2011). In particular, the construction of the questionnaires adopted conceptualization of strategic processing as a two-component construct with one as cognitive strategic processing and the other being metacognitive strategic processing.

The construction of the questionnaires followed the following procedure. First, a comprehensive literature search on language learning and use strategy questionnaires, in particular, language testing strategy questionnaires of the four skills, were gathered. Second, according to the nature of the sample Flyers tests and considering the age of the participants, items which were relevant to the Flyers test and were appropriate for the young ELLs were selected. As the majority of the existing questionnaires target on the adult ELLs or adult test-takers, the language of the selected items was modified so that it was comprehensible to young children. Third, the initial compilation of the items was discussed among three experts in language testing to evaluate the appropriateness of each item. After three rounds of discussions, the items which were unrelated to the Flyers tests or were inappropriate for children were deleted. The retained items were translated into Chinese by the researcher, who is an Australian National Accreditation Authority for Translators and Interpreters (NATTI) Certified English and Chinese translator. The draft Chinese questionnaires were double checked by another NATTI certified English and Chinese translator and then sent to a young Chinese ELL, who had similar age and English learning background as the participants, to check the comprehensibility of the items. Using the young learner’s feedback, the unclear and ambiguous Chinese expressions were revised. The final version of the Chinese questionnaires was used for the data collection. The questionnaires were on five-point Likert scales (1-Never, 2-Rarely, 3-Sometimes, 4-Usually, 5-Always). The items were written in simple past tense and the participants were asked to respond to the questionnaires by recalling the strategic processing in completing corresponding sections of the Flyers test.

The Strategic Processing in Listening Questionnaire had 15 items: 8 items (1, 2, 3, 4, 8, 9, 10, and 14) assessed students’ cognitive processing in listening, including listening for key words and overall meaning, comprehending, relating, predicting, and translating; 7 items (5, 6, 7, 11, 12, 13, and 15) evaluated students’ metacognitive processing in listening, including planning, evaluating, and monitoring.

The Strategic Processing in Reading and Writing Questionnaire consisted of 18 items: 10 items (4, 5, 6, 7, 8, 9, 10, 11, 12, and 13) examined cognitive processing in reading and writing, such as comprehending, retrieving, memorizing; and 8 items (1, 2, 3, 14, 15, 16, 17, and 18) assessed metacognitive processing in reading and writing, including, planning, evaluating, and monitoring.

For the Strategic Processing in Speaking Questionnaire, there were 15 items: 10 items (3, 4, 5, 6, 7, 8, 9, 12, 13, and 15) investigated cognitive processing in speaking, including paraphrasing, using intonation, and gesturing; and 5 items (1, 2, 10, 11, and 14) were concerned with metacognitive processing, such as planning, evaluating, and monitoring.

### Data Collection Procedure

Before the data collection, the participants and their accompanying parents were informed about the voluntary nature of the study. They were asked to sign a written consent form and only those returned a signed consent were included in the data collection. The data collection was undertaken in a Flyers test immediately after the test-takers completed the test. The questionnaires of strategic processing of listening, and reading and writing were distributed and collected in groups upon completion of the two sections. The collection of strategic processing of speaking was conducted individually immediately following each test-taker’s speaking test. The data collection was organized and supervised by the staff working in the Cambridge test center. To minimize potential problems arising from students with reading difficulties, the staff read each item in Chinese for the participants.

### Data Analysis

Both descriptive and inferential statistical analyses were performed. In order to answer RQ1 – the nature of strategic processing in listening, reading and writing, and speaking, confirmatory factory analyses (CFAs) were performed because the design of the questionnaires was adapted from well-established questionnaires with pre-specified structures. For evaluating CFA models, the general procedures proposed by Jöreskog and Sörbom (2005) and Kline (2005) were followed, and the following goodness-of-fit indices were considered as primary indicators of fit of the models: the Tucker-Lewis Index (TLI, Tucker and Lewis, 1973), the Comparative Fit Index (CFI, Bentler, 1990), and the root-mean-square error of approximation (RMSEA, Browne and Cudeck, 1993). According to Bentler (1990) and Hu and Bentler (1999), the values of TLI and CFI higher than 0.90 is generally considered an acceptable fit to the data. For RMSEA, Browne and Cudeck (1993) suggest that the values below 0.06 are indicative a good fit between the hypothesized model and the observed data. Besides these fit statistics, the factor loadings of items in corresponding scales were also checked to make sure that they were greater than 0.30. The Cronbach’s alpha reliability analyses were also performed to examine the internal reliability of the scales. The CFAs were performed in Mplus version 7.2. Once the constructs were validated using the CFAs, the *M* scores of the sub-categories of the constructs were calculated and used in the subsequent analyses.

For RQ2, Pearson correlation analyses were employed. To provide answers to RQ3, the participants were first grouped into high-, moderate-, and low-performing test-takers using approximately 33 and 67% percentile of their total shields. Then three separate 2 × 3 mixed factorial MANOVAs and *post hoc* analyses were conducted for three sections of the Flyers test using type of strategic processing as a within-subject independent variable, levels of test performance as a between-subject independent variables, and frequency of cognitive and metacognitive processing as dependent variables. Data analyses for RQ2 to RQ3 were conducted in SPSS 22.

## Results

### Results of Research Question 1 – The Nature of Strategic Processing in the Flyers Test

For the structure of strategic processing in listening, items 5 and 6 were deleted due to their low item-total correlations. The remaining 13 items retained a two-factor solution with items 1, 2, 3, 4, 8, 9, 10, 14 representing a cognitive scale (α = 0.81) and items 7, 11, 12, 13, 15 being a metacognitive scale (α = 0.80). The two-factor model yielded good fit (χ^2^ = 76.24, TLI = 0.96, CFI = 0.94, RMSEA = 0.05). All the factor loadings of items were above 0.46.

In terms of the nature of strategic processing in reading and writing, item 2 was removed due to its low item-total correlation. The rest of 17 items produced a two-factor model with items 4, 5, 6, 7, 8, 9, 10, 11, 12, 13 being a cognitive factor (α = 0.86), and items 1, 3, 14, 15, 16, 17, 18 being a metacognitive factor (α = 0.82). The two-factor solution revealed good fit between the hypothesized model and the data (χ^2^ = 151.42, TLI = 0.96, CFI = 0.95, RMSEA = 0.05), with all the factor loadings above 0.46.

With regard to the construct of strategic processing in speaking, item 3 was eliminated due to its low item-total correlation. The rest of 14 items also resulted in two subscales with items 4, 5, 6, 7, 8, 9, 12, 13, 15 representing a cognitive component (α = 0.81), and items 1, 2, 10, 11, 14 being a metacognitive component (α = 0.75). The fit statistics showed an acceptable fit (χ^2^ = 92.25, TLI = 0.96, CFI = 0.93, RMSEA = 0.06), with all the factor loadings above 0.32. The retained items, their factor loadings on the respective scales, and the scale reliability are summarized in **Table 3**.

**Table 3:** Items, factor loadings, and reliability for the questionnaires.

Scales	Items	Factor loadings	
Cognitive in listening	I listened for overall meaning.	0.59	
(8 items) α = 0.81	I thought of what I might know about the audio files by using the pictures on the test paper.	0.65	
	I used sound effects and tone of the speaker’s voice to help me guess the meaning of words.	0.47	
	As I was listening, I used words that I recognized to help me guess the meaning of unknown words.	0.51	
	As I listened, I related what I was hearing to what I had understood earlier.	0.62	
	I used the questions asked in the test to help me predict what I would hear.	0.59	
	As I listened, I focused on the main words.	0.59	
	As I was listening, I tried to translate the English into Chinese in my mind.	0.57	

Metacognitive in	When I was having trouble understanding, I told myself that I would manage and would do fine.		0.57
listening	When I had trouble understanding, I paid more attention and focused harder.		0.74
(5 items) α = 0.80	When I had trouble understanding, I kept on listening because I expected to understand more later.		0.62
	When my mind wandered, I recovered my concentration right away.		0.70
	When I was listening, I had a good idea when I understood something and when I did not.		0.67

Cognitive in reading	I tried to understand the relationships between ideas in the text.	0.64	
and writing	I tried to understand the content of the text without reading every word.	0.51	
(10 items) α = 0.86	I predicted what was going to happen next while I was reading the text.	0.50	
	I translated the texts into Chinese.	0.50	
	I summarized the main information in the text.	0.57	
	I related the information from the text to my prior knowledge and experience.	0.72	
	I reread texts or tasks several times when I felt I did not understand them.	0.69	
	I knew which information was more or less important in reading.	0.73	
	I guessed meanings of unknown words using context clues.	0.58	
	I tried to understand the relationships between ideas in the text.	0.64	

Metacognitive in	I planned essential steps needed to complete the reading test.		0.61
reading and writing	I knew what to do if my intended plans did not work efficiently while completing this reading test.		0.71
(7 items) α = 0.82	When I lost concentration in reading I tried to pay more attention and focus harder.		0.66
	I knew when I should read more quickly or carefully.		0.63
	I double-checked my reading comprehension.		0.73
	I immediately corrected any misunderstandings I had in the reading and writing paper when found.		0.66
	I knew how much of the reading and tasks remained to be done.		0.47

Cognitive in speaking	I tried to sound every word clearly when speaking.	0.70	
(9 items) α = 0.81	When I could not think of English words to say a message, I made the idea simpler.	0.57	
	When I spoke, I tried not to translate Chinese to English word-for-word.	0.51	
	When I could not think of a word during speaking, I used gestures.	0.33	
	I made up new words if I did not know the right ones.	0.38	
	I paid attention to what the examiner asked.	0.66	
	When I could not understand the examiner’s questions, I asked the examiner to explain in different words.	0.46	
	I knew which information was more or less important to say.	0.60	
	I made sure I used correct intonation when speaking.	0.62	

Metacognitive in	I planned what to say in my mind before I began speaking.		0.65
speaking	I made sure the responses I made answered questions asked by the examiner.		0.63
(5 items) α = 0.75	I tried to make sure that I did not make grammatical mistakes when I spoke.		0.71
	I encouraged myself when I felt nervous to speak.		0.58
	When I did not hear something clearly, I knew what to do next to respond.		0.55


**Table 4** presents descriptive statistics of the scales, including min., max., *M*s, and *SD*s. Consistently in the three sections of the Flyers test, our young ELLs exhibited dual-components structure of strategic processing, which was similar to that of the adult test-takers.

**Table 4:** Descriptive statistics of the scales of the Strategic Processing Questionnaires.

Section	Minimum	Maximum	*M*	*SD*
Cognitive in listening	1.00	5.00	3.75	0.86
Metacognitive in listening	1.00	5.00	4.23	0.82
Cognitive in reading and writing	1.00	5.00	3.90	0.80
Metacognitive in reading and writing	1.00	5.00	4.01	0.85
Cognitive in speaking	1.00	5.00	3.67	0.85
Metacognitive in speaking	1.00	5.00	3.94	0.90


### Results of Research Question 2 – Relations Between Cognitive, Metacognitive Strategic Processing, the Shields in Each Section, and the Total Shields

The results of correlation analyses are presented in **Table 5**, which shows that within each section of the test, cognitive and metacognitive processing are consistently and positively associated, and the strength of the correlation coefficients are moderate (listening: *r* = 0.67, *p* < 0.01; reading and writing: *r* = 0.79, *p* < 0.01; speaking: *r* = 0.73, *p* < 0.01). Across the three sections of the test, the correlations of cognitive strategic processing in one skill also positively and moderately related to that in the other skills (*r*s range between 0.58 and 0.70), and this pattern was also applicable to the correlations among metacognitive strategic processing in different skills (*r*s range between 0.63 and 0.73).

**Table 5:** Results of correlation analyses.

Variables	Meta in	Cog.	Meta	Cog in	Meta in	Listening	RW	Speaking	Total
	listening	in RW	in RW	speaking	speaking	shields	shields	shields	Shields
Cog. in listening	0.67^∗∗^	0.70^∗∗^	0.66^∗∗^	0.58^∗∗^	0.63^∗∗^	0.01	0.11	-0.08	0.27^∗∗^
Meta in listening	—	0.66^∗∗^	0.73^∗∗^	0.52^∗∗^	0.63^∗∗^	0.05	0.09	-0.09	0.29^∗∗^
Cog. in RW	—	—	0.79^∗∗^	0.66^∗∗^	0.66^∗∗^	0.11	0.15	-0.07	0.39^∗∗^
Meta in RW	—	—	—	0.66^∗∗^	0.68^∗∗^	0.11	0.22^∗^	-0.06	0.40^∗∗^
Cog. in speaking	—	—	—	—	0.73^∗∗^	0.09	0.21^∗^	-0.08	0.40^∗∗^
Meta in speaking	—	—	—	—	—	0.13	0.21^∗^	-0.02	0.56^∗∗^
Listening shields	—	—	—	—	—	—	0.58^∗∗^	0.35^∗∗^	0.73^∗∗^
RW shields	—	—	—	—	—	—	—	0.16	0.75^∗∗^
Speaking shields	—	—	—	—	—	—	—	—	0.15


*^∗^*p* < 0.05, ^∗∗^*p* < 0.01. Cog., cognitive processing; Meta, metacognitive processing; RW, reading and writing.*

With regard to the strategic processing and the test performance in each section, it was found that metacognitive processing in reading was positively and weakly related to the reading shields (*r* = 0.22, *p* < 0.05). Somewhat out of expectation that even though strategic processing in speaking did not have significant correlations with the speaking shields, they positively and weakly correlated with the shields in reading (cognitive: *r* = 0.21, *p* < 0.05; metacognitive: *r* = 0.21, *p* < 0.05).

In terms of the relationship between strategic processing and the total shields, the results showed that metacognitive strategic processing in listening (*r* = 0.29, *p* < 0.01) had slightly stronger association with the test performance than that between cognitive strategic processing in listening and the total shields (*r* = 0.27, *p* < 0.01). A *z*-test showed that such difference was not significant (*z* = -0.03, *p* = 0.76). In the reading and writing section, the value of correlation coefficients between cognitive strategic processing and the total shields (*r* = 0.39, *p* < 0.01) was similar to that between metacognitive strategic processing and the total shields (*r* = 0.40, *p* < 0.01). However, in the speaking section, the metacognitive strategic processing (*r* = 0.56, *p* < 0.01) had stronger association with the total shields than that between cognitive strategic processing and the total shields (*r* = 0.40, *p* < 0.01). The *z*-test revealed that such difference was significant (*z* = -2.98, *p* < 0.01). The variance of the total shields explained by the cognitive and metacognitive strategic processing in different sections of the test varied: ranging from 7.29% (i.e., cognitive processing in listening) to 31.36% (i.e., metacognitive processing in speaking).

### Results of Research Question 3 – The Effects of Strategic Processing Type and Levels of Test Performance on Cognitive and Metacognitive Strategic Processing

**FIGURE 1:**
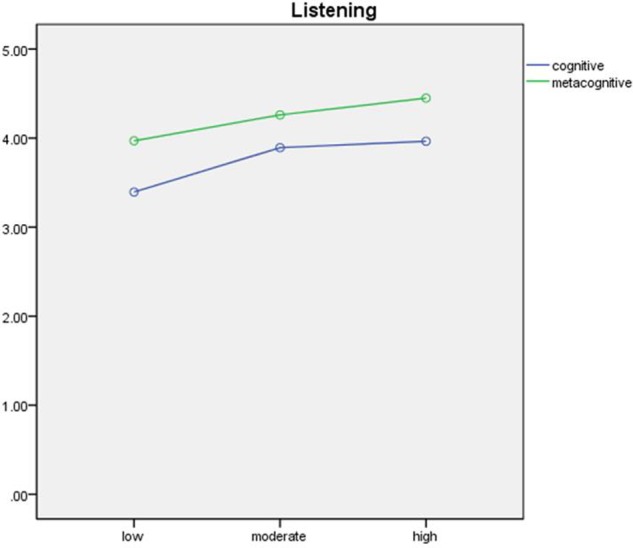
Visual display of the results of the mixed MANOVA for listening.

**FIGURE 2:**
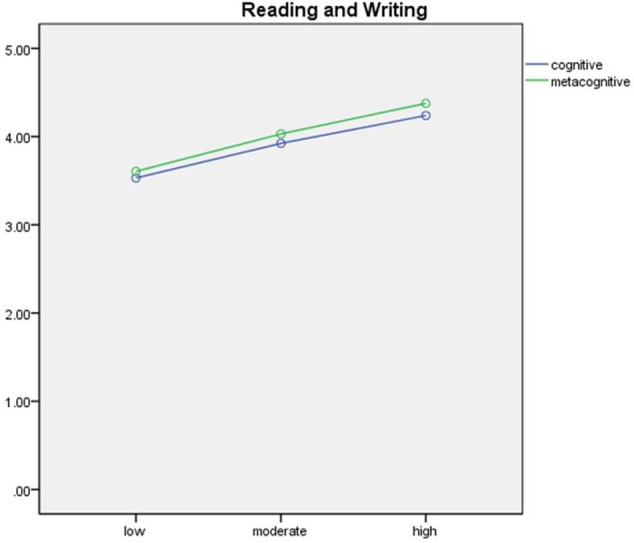
Visual display of the results of the mixed MANOVA for reading and writing.

**FIGURE 3:**
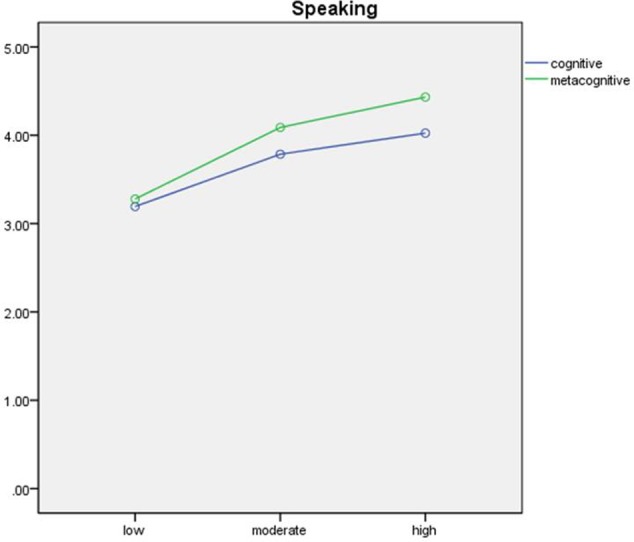
Visual display of the results of the mixed MANOVA for speaking.

The results of the mixed MANOVAs for listening, reading and writing, and speaking are presented in **Table 6** and are visually displayed in **Figures 1**–**3**.

**Table 6:** Results of the mixed MANOVAs.

	Variables *M* (*SD*)				*F*		
		
	Overall	High	Moderate	Low	Type	Level	Interaction
	*N* = 138	*N* = 49	*N* = 43	*N* = 46			
	*Listening*				65.90^∗∗^	6.38^∗∗^	1.01
Cog.	3.75 (0.86)	3.96 (0.75)	3.89 (0.86)	3.89 (0.86)			
Meta	4.23 (0.82)	4.45 (0.75)	4.26 (0.81)	3.97 (0.86)			
	*Reading and writing*				5.19^∗^	11.42^∗∗^	0.15
Cog.	3.90 (0.80)	4.24 (0.69)	3.92 (0.79)	3.53 (0.78)			
Meta	4.01 (0.85)	4.38 (0.73)	4.03 (0.79)	3.61 (0.85)			
	*Speaking*				24.29^∗∗^	24.62^∗∗^	3.20^∗^
Cog.	3.67 (0.85)	4.02 (0.81)	3.79 (0.74)	3.19 (0.79)			
Meta	3.94 (0.90)	4.43 (0.67)	4.09 (0.71)	3.28 (0.89)			


*^∗^*p* < 0.05, ^∗∗^*p* < 0.01. Cog., cognitive processing; Meta, metacognitive processing.*

**Table 6** showed that in listening, type was a significant main within-subjects effect, *F*(1,135) = 65.90, *p* < 0.01, $\eta_{p}^{2}$ = 0.33. The Chinese young ELLs operated metacognitive strategic processing (*M* = 4.23, *SD* = 0.82) more frequently than cognitive strategic processing (*M* = 3.75, *SD* = 0.86) in listening. In terms of the between-subjects effect, levels of test performance also had a significant effect on cognitive and metacognitive processing, *F*(2,135) = 6.38, *p* < 0.01, $\eta_{p}^{2}$ = 0.09. The Bonferroni *post hoc* analyses showed that high-performing test-takers (*M* = 3.96, *SD* = 0.75) used cognitive strategies in listening significantly more frequently than the moderate-performing test-takers (*M* = 3.89, *SD* = 0.86), which in turn used more frequently than the low-performing ELLs (*M* = 3.39, *SD* = 0.87). For metacognitive processing in listening, the only significant difference was between high- (*M* = 4.45, *SD* = 0.45) and low-achieving test-takers (*M* = 3.97, *SD* = 0.86). The interaction effect between type of strategic processing and levels of test performance was not significant, *F*(2,135) = 1.01, *p* = 0.37, $\eta_{p}^{2}$ = 0.02.

For the reading and writing section, the results show that type was also significant, *F*(1,135) = 5.19, *p* < 0.05, $\eta_{p}^{2}$ = 0.04. In the process of completing reading and writing section, the test-takers self-reported that using metacognitive strategies (*M* = 4.01, *SD* = 0.85) significantly more frequently than cognitive strategies (*M* = 3.90, *SD* = 0.80). The levels of test-performance also exhibited a significant effect on the use of cognitive and metacognitive strategies, *F*(2,135) = 11.42, *p* < 0.01, $\eta_{p}^{2}$ = 0.16. *Post hoc* analyses demonstrated that all the pairwise comparisons between the three levels of test-takers in both cognitive and metacognitive strategy use were significant: consistently the high-ability test-takers (cognitive: *M* = 4.24, *SD* = 0.69; metacognitive: *M* = 4.38, *SD* = 0.73) reported using more cognitive and metacognitive strategies than the moderate-performing test-takers (cognitive: *M* = 3.92, *SD* = 0.79; metacognitive: *M* = 4.03, *SD* = 0.79), which again used more than the low-ability counterparts (cognitive: *M* = 3.53, *SD* = 0.78; metacognitive: *M* = 3.61, *SD* = 0.85). The interaction did not reach significant, *F*(2,135) = 0.15, *p* = 0.86, $\eta_{p}^{2}$ = 0.00.

In terms of strategic processing in speaking, it was found that type, *F*(1,135) = 24.29, *p* < 0.01, $\eta_{p}^{2}$ = 0.15; levels of test performance, *F*(2,135) = 24.62, *p* < 0.01, $\eta_{p}^{2}$ = 0.27; and the interaction of the two, *F*(2,135) = 3.20, *p* < 0.05, $\eta_{p}^{2}$ = 0.05, were all significant. In the speaking, the test-takers were significantly involved more frequently in the metacognitive strategic processing (*M* = 3.94, *SD* = 0.90) than in the cognitive strategic processing (*M* = 3.67, *SD* = 0.85). The Bonferroni *post hoc* analyses showed that while the high-performing learners (*M* = 4.02, *SD* = 0.81) operated more frequently than the moderate-performing counterparts (*M* = 3.79, *SD* = 0.74), who again showed adopting more cognitive strategic behaviors than the low-performing children (*M* = 3.19, *SD* = 0.79). All the pairwise comparison of metacognitive strategic processing in speaking was significantly different among the three groups of ELLs: the high-performing ELLs (*M* = 4.43, *SD* = 0.67) used more metacognitive strategies than the moderate-performing ELLs (*M* = 4.09, *SD* = 0.71), who also employed more than the low-performing ELLs (*M* = 3.28, *SD* = 0.89).

Because of the significant interaction effect, the differences between cognitive and metacognitive strategic processing in the three groups were also explored using three separate paired *t*-tests. The results demonstrated that the difference of frequency of using metacognitive and cognitive strategies were significant among the high-performing [*t*(1,48) = -4.86, *p* < 0.01, Cohen’s *d* = -0.63] and moderate-performing ELLs [*t*(1,42) = -3.63, *p* < 0.01, Cohen’s *d* = -0.55], no such difference was found among the low-performing ELLs [*t*(1,45) = 0.87, *p* = 0.39, Cohen’s *d* = -0.14].

In summary, consistently across the three sections of the Flyers test, it was observed that our young Chinese test-takers were engaged more often in metacognitive strategic processing than cognitive strategic processing. However, low-performing students did not show any difference in using cognitive and metacognitive strategies in the speaking test. In addition, high-performing ELLs also reported using more cognitive and metacognitive strategies than their low-performing counterparts in the three sections of the test.

## Discussion

Across the three sections of the Flyers test, the results revealed that two latent factors of strategic processing: one cognitive and one metacognitive factor, among Chinese young ELLs. This seems to indicate that our child ELLs were aware of and were able to reflect upon the strategies they had employed in the test. The two-dimensionality of the strategic processing construct in listening, reading and writing, as well as speaking among the child ELLs resembled the nature of the strategic processing among the adult ELLs (e.g., Phakiti, 2003a,b). However, there has been inconsistency with regard to whether the cognitive and metacognitive strategic processing factors are constituted by multiple sub-categories respectively (e.g., Phakiti, 2003a,b, 2008a). For instance, Phakiti (2008a) reported that the cognitive processing factor comprised of comprehending, memorizing, and retrieving sub-factors, and the metacognitive strategic processing consisted of planning, monitoring, and evaluating. But these sub-factors was not be able to replicated in Phakiti (2003a,b) in which there were only a cognitive and metacognitive factor without identification of sub-factors. These seem to indicate that we cannot assume the nature of the strategic processing revealed from our sample to be representative of the nature of strategic processing among other child ELLs population. In order to further validate the structure of the strategic processing in different language use skills in language tests, more studies are needed with more diverse populations.

One of the reasons why the study did not purposefully make fine-tuned sub-categories for cognitive and metacognitive strategic processing in our questionnaires was related to the young age of the participants and the contents of the test items in the Flyers. Because our participants were children, they might not understand the ideas, such as retrieving and evaluating. Moreover, in order to reveal fine-tuned categories, more items on different sub-categories of cognitive and metacognitive processing would have to be added into the questionnaires. Potentially, this would lead to fatigue of the participants, who only have short period of concentration a their age. The reality was that our young test-takers had to spend almost one and a half hours to complete listening, reading, and writing parts of the test before responding to the questionnaires. If they had faced a large number of questionnaire items, they might not have answered the questionnaires completely, or even worse, they might not have agreed to participate the study voluntarily. When they had responded the questionnaires under the condition of being not mentally concentrated, they might not have understood the statements in the questionnaires properly after the intense language test, and hence, the responses would have not been reliable. Another reason for us not including a wider range of items on strategic processing was related to the contents of the tests. The selection of the items was carefully carried out according to the sample test items available online. As a result, some items pertaining to the complexity of strategic processing in the questionnaires for adult test-takers were not appropriate for our participants.

Our results that moderate and positive correlations (*r*s between 0.71 and 0.82) between cognitive and metacognitive strategic processing across the four skills corroborated with previous findings among adult test-takers in reading tests (e.g., Phakiti, 2003a,b, 2008a,b) and we found that the child test-takers’ cognitive and metacognitive strategic behaviors function in concert with each other, similar to what has been described by Phakiti (2003b) that cognitive and metacognitive strategic processing are “two interactive facets of the same mental process that do not occur independently of each other” (p. 48).

Furthermore, our results that both cognitive and metacognitive strategic processing in one section of the Flyers test are positively and moderately related to that in the other two sections seems to suggest that the test-takers who are able to operate strategic processing in listening, also tend to be strategic in handling reading, writing, and speaking tasks. While previous studies report that ELLs are able to transfer their strategic behaviors learnt from L1 to FL (e.g., van Gelderen et al., 2004, 2007; Stevenson et al., 2007; Han and Stevenson, 2008); our study seems to indicate that the ability to execute strategic processing in one skill in a FL is also transferable to other skill(s) in that FL.

In terms of the variance of the test performance explained by the young ELLs’ strategic processing, surprisingly it appears that strategic processing in one skill is much more strongly related to the overall test performance than the performance in that skill. In fact, within a language skill, we only observed that metacognitive strategic processing and reading and writing shields had significant relationship. Such result might be largely attributable to the written mode of the reading and writing section, which might enable the test-takers to implement metacognitive strategies more successfully compared to in the listening and speaking sections. The audio and oral modes of the listening and speaking might not give test-takers sufficient time for metacognitive strategies to be carried out successfully. This finding seems to align with the Compensatory Encoding Model (CEM) (Walczyk, 2000), even though the construction of the CEM aims at explaining how readers use metacognitive strategies to compensate for inefficient processing in reading in order to achieve good level of comprehension. Nonetheless, it postulates for metacognitive strategies to operate successfully as a compensatory mechanism for processing inefficiency, sufficient time is necessary (Walczyk et al., 2001, 2007). This speculation could be further evident in our results in the speaking section, where no difference was found between the cognitive and metacognitive strategies only among the low-achieving students, who presumably took longer time to convert their conceptual ideas into English, possibly impeding their execution of metacognitive strategies. On the other hand, the moderate- and high-achieving students might have achieved a level of processing which allowed them to use metacognitive strategies in the online information processing during the spoken mode, and they indeed adopted more metacognitive strategies than cognitive strategies in speaking.

The predictive power of our test-takers’ self-reported use of cognitive and metacognitive strategies in listening, reading and writing, and speaking to the total shields also varied, from around 7 to 31%. This range of the variance explained seems to be practical and meaningful, because strategic competence is only one of components of CLA, which may impact on language test performance (Bachman and Palmer, 1996, 2010). Admittedly, linguistic knowledge would play a predominant role than the strategic competence on how well-learners can achieve in language tests.

Our MANOVA results that across the three sections of the Flyers test, the test-takers adopted metacognitive strategies more frequently than cognitive strategies might be due to the formality of the test. As the participants sat an international standardized language test; they might have carefully planned each step (planning strategies) in the test, as well as have constantly monitored and assessed if their plans worked out or not (monitoring and evaluating strategies). In the case that previous plans did not function as desired, they might have modified previous strategies and actively made justifications or have taken different routes or procedures to tackle the test items. As strategic behaviors tend to fluctuate from one situation to another, it was speculated that the learners might not have adopted as many metacognitive strategies as in this international standardized test, if they had sat a test given by their English teachers, or if they had used English in a non-testing situation. However, such speculation should be testified in the future research by comparing strategic processing in testing versus non-testing situations.

With regard to the influence of levels of test-performance on strategic processing, the findings demonstrated that the high-achieving learners adopted both cognitive and metacognitive strategies more frequently than low-achieving test-takers. These were similar to the results obtained among adult test-takers in the reading tests (Phakiti, 2003b). Our study extended the strategic processing in the reading skill to other skills, including writing, listening, and speaking. Three possible explanations could explain the differences of strategic processing between high- and low-performing students. As indicated by the linguistic threshold hypothesis (e.g., Clarke, 1979; Cummins, 1979; Alderson, 1984), the low-performing test-takers might not have a certain level of English linguistic knowledge, which could short-circuit their use of strategies in the test. It could also be plausible that the high-achieving test-takers were more efficient at processing linguistic codes, so that their working memory was able to be freed, allowing strategic processing was more successfully executed than low-performing students during the test, as suggested by the processing efficiency hypothesis (e.g., Koda, 1996; Segalowitz, 2000). Another possible explanation for the more strategic behaviors by the high-achieving students could be that they had a relatively large strategic repertoire to draw upon (i.e., strategic knowledge) than low-achieving peers. However, as the study did not measure strategic knowledge, which is stable, enduring, and trait like dispositions (Phakiti, 2003a,b, 2006, 2008a,b; Westby, 2004; Han and Stevenson, 2008; Han, 2012; Flavell, 2016; Wang and Han, 2017), it is hard to verify such speculation. To explicate the complicated relationship between strategic knowledge, strategic processing, and language performance in language tests among young children, future studies may consider measuring both trait-like knowledge of strategies and context-specific strategic processing.

### Implications

#### Theoretical Implications

Theoretically, the current study contributed to the language testing literature on strategic processing among young children, which is an under researched area compared with relatively large number of studies with adult test-takers. The study shows that young children are conscious about strategies they have used and the strategic processing is a two-dimensional construct, encompassing cognitive and metacognitive strategic processing, consistently across all the four skills: listening, reading and writing, and speaking. Language testing research with young children is essential and important because more and more people start to learn English at a younger age worldwide, and more types of English proficiency tests are designed and available for young ELLs in order to test their English language ability and to motivate them to learn English. Although the study only focused on ELLs from a single L1 background – Chinese, which may limit the generalizability of the results, it may serve as a springboard for more studies to be carried out in the area of test-taking strategies among young ELLs from diverse L1 backgrounds.

#### Practical Implications

As has been shown in our study that strategic processing in listening, reading and writing, as well as speaking all have positive and moderate association with the total shields, the task remained for Cambridge English trainers and instructors is how to equip young ELLs with such strategic processing apart from help them acquire linguistic knowledge. Teachers may wish to teach cognitive and metacognitive strategies through explicit instruction, which will likely to result in a richer strategic knowledge for young learners, as it is reasonable to assume that young ELLs’ strategic repertoire is still in the development and not yet complete. In particular, teachers can raise young children’s awareness of the usefulness of using metacognitive strategies, including planning, monitoring, evaluating, and making adjustment, as using metacognitive strategies seems to be more strongly related to the test performance. For instance, teachers may model the process of using metacognitive strategies, such as how to conceive a good and practical plan to complete language tests beforehand, how to evaluate and monitor whether the plan works or not, and how to update the plans and strategic behaviors by trying different kinds of strategies, or combining a number of strategies in order to solve the problem at hand. Teachers can also train young ELLs’ metacognitive strategies in each language skill separately. In listening, for example, training students’ selective attention on key words, phrases, and linguistic markers when listening will be useful for them to grasp the main ideas of speech. In the reading, constantly monitoring and checking if their comprehension makes sense or not is useful for children to stay focused during reading. In a speaking test, teachers can train students how to quickly make plans of what and how to say things, and how to arrange the key points in a logical order in their minds. It is hoped that these examples may trigger teachers to design some age-appropriate strategic training activities which can be embedded in language teaching to young ELLs.

## Ethics Statement

This study was carried out in accordance with the recommendations of ‘National Statement on Ethical Conduct in Human Research, the Human Research Ethics Committee of the University of Sydney’ with written informed consent from all subjects and their parents. All subjects and their parents gave written informed consent in accordance with the Declaration of Helsinki. The protocol was approved by ‘the Human Research Ethics Committee of the University of Sydney.’

## Author Contributions

The author confirms that she contributed to this paper by: contributing substantially to the conception of the work; and the acquisition, analysis, and interpretation of the data; drafting the work and revising it critically for important intellectual content; approving the final version of the paper to be published and agreeing to be accountable for all aspects of the work in ensuring that questions related to the accuracy or integrity of any part of the work are appropriately investigated and resolved.

## Conflict of Interest Statement

The author declares that the research was conducted in the absence of any commercial or financial relationships that could be construed as a potential conflict of interest.

## Footnotes

1 http://www.cambridgeenglish.org/exams-and-tests/flyers
